# Hope Against Resistance: In Vitro Activity of the Ceftazidime-Avibactam and Aztreonam Combination Against Colistin-Resistant Klebsiella pneumoniae

**DOI:** 10.7759/cureus.110850

**Published:** 2026-06-14

**Authors:** Munmun Mutha, Parimala Subramani, Raveesha A

**Affiliations:** 1 Microbiology, Sri Devaraj Urs Medical College, Kolar, IND; 2 General Medicine, Sri Devaraj Urs Medical College, Kolar, IND

**Keywords:** aztreonam (atm), carbapenem-resistant klebsiella pneumoniae, ceftazidime-avibactam, colistin-resistant k. pneumoniae, drug synergism

## Abstract

Background

Carbapenem-resistant *Klebsiella pneumoniae* (CRKP) represents a major global health threat. Rising colistin resistance in CRKP further restricts therapeutic options. With no newer antibiotics in the pipeline, the combination of ceftazidime-avibactam (CZA) and aztreonam (ATM) has emerged as a rational strategy to overcome complex β-lactamase-mediated resistance.

Aim

The aim of this study was to determine the in vitro susceptibility and synergy rates of CZA + ATM among colistin-resistant CRKP isolates.

Methods

A prospective laboratory study including 17 colistin-resistant CRKP isolates was conducted. Synergy testing was performed using the disk elution method in cation-adjusted Mueller-Hinton broth (CA-MHB), as per the latest CLSI guidelines.

Results

Notably, 12 of 17 (70.6%) isolates demonstrated synergistic susceptibility to CZA + ATM. Three isolates (17.6%) were susceptible to CZA alone, and the remaining two (11.8%) were susceptible to ATM alone.

Conclusion

CZA + ATM demonstrates remarkable in vitro efficacy against colistin-resistant CRKP and is a notable carbapenem- and colistin-sparing treatment alternative.

## Introduction

Carbapenem-resistant *Klebsiella pneumoniae* (CRKP) has been emerging as a major challenge in modern clinical practices, particularly within hospitals where antibiotic pressure and vulnerable patient populations facilitate the persistence and spread of multidrug-resistant organisms [1]. Infections caused by CRKP are frequently associated with unfavorable clinical outcomes, including increased morbidity and mortality, prolonged hospitalization, and considerable healthcare expenditure. The growing burden of these infections has led to global concern, prompting the World Health Organization (WHO) to declare carbapenem-resistant Enterobacterales as critical priority pathogens requiring urgent attention for the development of novel antimicrobial therapies [2].

The development of carbapenem resistance in *K. pneumoniae* is largely driven by the acquisition and dissemination of carbapenemase enzymes. Among these, New Delhi metallo-β-lactamase (NDM), OXA-48-like enzymes, and *K. pneumoniae* carbapenemase (KPC) represent the most significant groups. NDM, in particular, is highly prevalent in the Indian subcontinent and is frequently associated with rapid horizontal gene transfer, contributing to widespread dissemination [3]. These enzymes confer resistance by hydrolyzing a broad range of β-lactam antibiotics, including carbapenems, thereby significantly narrowing the available therapeutic options. Furthermore, the coexistence of additional resistance factors such as extended-spectrum β-lactamases (ESBLs), AmpC β-lactamases, porin loss, and efflux pump overexpression often results in highly complex resistance phenotypes, which further limit treatment efficacy.

Polymyxins, especially colistin, have traditionally been considered agents of last resort for managing infections caused by carbapenem-resistant organisms. However, their clinical utility has been increasingly compromised due to both safety concerns and the emergence of resistance. Colistin-associated nephrotoxicity has restricted its role in treating multidrug-resistant organisms, while the increase in resistance mediated by chromosomal mutations and plasmid-borne *mcr* genes has significantly reduced its effectiveness [4]. Recent Indian surveillance studies have reported colistin resistance rates among CRKP ranging from 8% to 20%, highlighting the increasing therapeutic challenge posed by these isolates, as it leaves clinicians with limited and often suboptimal treatment options, thereby adversely affecting patient outcomes [5].

To overcome these limitations, recent advances in antimicrobial therapy have focused on β-lactam/β-lactamase inhibitor combinations designed to combat these specific resistance mechanisms. Ceftazidime-avibactam (CZA) is one such combination that has demonstrated potent activity against organisms producing Ambler class A β-lactamases, including KPC, as well as class C and class D enzymes such as OXA-48 carbapenemases [6]. Despite its broad activity, CZA lacks efficacy against metallo-β-lactamases (MBLs), which limits its use in regions where MBLs predominate. In contrast, aztreonam (ATM), a monobactam antibiotic, is inherently resistant to hydrolysis by MBL enzymes but is susceptible to degradation by other β-lactamases, particularly ESBLs and AmpC enzymes [7].

The combination of CZA with ATM has therefore been proposed as a rational therapeutic approach aimed at overcoming these complementary limitations. In this regimen, avibactam inhibits serine β-lactamases that would otherwise inactivate ATM, thereby preserving its activity against MBL-producing organisms. Effective targeting of pathogens that simultaneously harbor several resistance mechanisms is made possible by this synergistic interaction, which is frequently seen in multidrug-resistant *K. pneumoniae*. This combination may provide major microbiological and therapeutic benefits in infections caused by extensively drug-resistant Enterobacterales [8].

Despite these promising developments, there remains a relative lack of data evaluating the effectiveness of this combination specifically against colistin-resistant *K. pneumoniae*, particularly in India, where NDM-mediated resistance is highly prevalent. The coexistence of polymyxin resistance with carbapenemase production further complicates treatment decisions and highlights the need for locally generated evidence to guide clinical practice.

In this context, this study was undertaken to evaluate the in vitro antimicrobial activity and synergistic potential of CZA and ATM against carbapenem-resistant, colistin-resistant *K. pneumoniae* isolates obtained from various clinical samples. This study seeks to enhance the current evidence base for combination-based therapeutic options in the management of challenging CRKP infections by evaluating the degree of synergy and patterns of susceptibility.

## Materials and methods

This study was designed as a prospective, laboratory-based observational study conducted at the Central Diagnostic Laboratory Services (CDLS) of R.L. Jalappa Hospital, Kolar, India. A total of 334 CRKP isolates were screened. All non-duplicate carbapenem- and colistin-resistant *K. pneumoniae* isolates recovered during the study period were included, yielding a total of 17 isolates.

Carbapenem resistance was detected by routine antimicrobial susceptibility testing according to CLSI guidelines. Colistin susceptibility testing was performed using broth microdilution (BMD), which is the CLSI-recommended reference method.

A total of 17 non-duplicate colistin-resistant CRKP isolates were included in the analysis. Synergy testing between CZA and ATM was performed using the disk elution method in cation-adjusted Mueller-Hinton broth (CA-MHB).

For evaluation of synergism, four sterile test tubes containing 5 mL of CA-MHB were labeled as CZA 10/4 µg, ATM 30 µg, CZA + ATM (combination), and GC (growth control). Appropriate antibiotic disks were added to the respective tubes. The tubes were gently vortexed to facilitate elution of antimicrobial agents from the disks into the broth and allowed to stand at room temperature for approximately 30 minutes to ensure adequate diffusion.

A standardized bacterial inoculum equivalent to 0.5 McFarland turbidity (approximately 1.5 × 10⁸ CFU/mL) was prepared and used for testing. The GC tube contained only the test organism without any antibiotic disks to confirm viability and adequate growth conditions.

After inoculation, the tubes were gently vortexed again at a slow speed to ensure uniform distribution of the inoculum and antibiotics. All tubes were then incubated aerobically at 37°C for 16-18 hours. The presence of a clear solution in the test tube indicated susceptibility to the antibiotic, whereas visible turbidity indicated resistance. These results were interpreted according to CLSI guidelines, 2025 [9].

## Results

**Figure 1:**
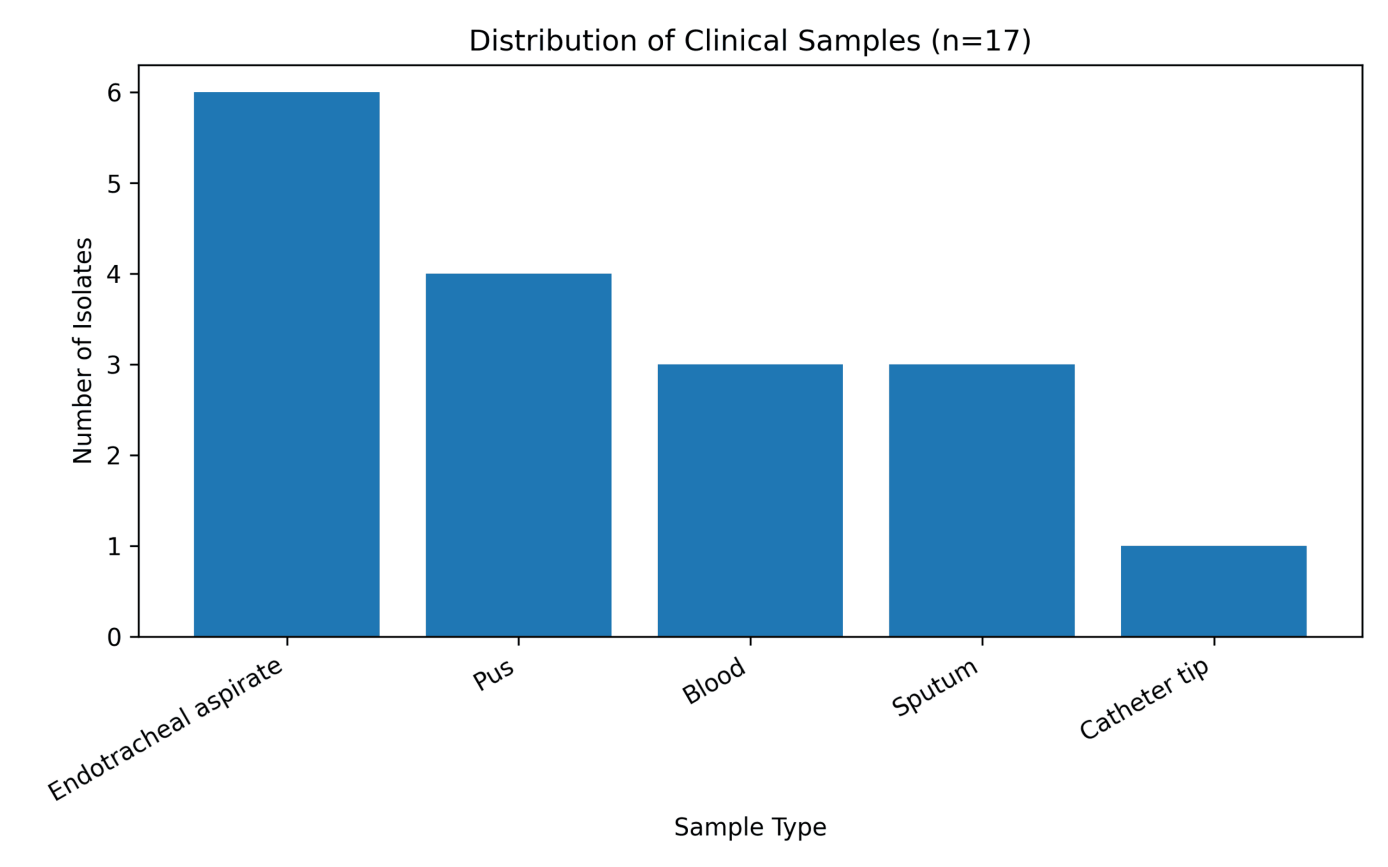
Distribution of colistin-resistant isolates by sample type (n = 17)

Most samples were respiratory, predominantly endotracheal aspirates and sputum, followed by pus and blood (Figure 1). Respiratory infections were the most common clinical presentation, including bronchopneumonia, aspiration pneumonia, ventilator-associated pneumonia, and respiratory failure, while other conditions such as sepsis, necrotizing fasciitis, meningoencephalitis, and wound- or fracture-related infections were less frequent. Neonatal cases presented with respiratory distress and sepsis.

All patients received tigecycline-based therapy. Tigecycline monotherapy was administered in 13 cases, while combination therapy was used in four cases: tigecycline with cotrimoxazole (n = 1), cefoperazone-sulbactam (n = 1), and fluconazole (n = 1).

Outcomes showed that 13 patients (76.5%) were discharged, while four patients (23.5%) died. Mortality was primarily observed in patients with severe systemic illness, including septic encephalopathy, acute respiratory distress syndrome, ventilator-associated pneumonia, and septic shock, often in the presence of significant comorbidities (Table 1).

**Table 1:** Demographic and clinical characteristics of colistin-resistant isolates HTN: hypertension; CVA: cerebrovascular accident; FTP: fronto-temporo-parietal

S. no.	Sex/Age	Specimen	Diagnosis	Co-morbidity	Treatment	Outcome
1	40/M	Pus	Right proximal femur fracture	None	Tigecycline + Cotrimoxazole	Discharged
2	76/F	Pus	Wound over Rt. Foot	Type II DM	Tigecycline + Cefoperazone sulbactam	Discharged
3	77/M	Endotracheal aspirate	Acute meningoencephalitis, Septic encephalopathy	HTN Type II DM	Tigecycline	Died
4	1 day/M	Blood	Respiratory distress secondary to birth asphyxia, Hepatic encephalopathy, Sepsis	None	Tigecycline + Fluconazole	Discharged
5	48/F	Endotracheal aspirate	CVA/FTP craniectomy	None	Tigecycline	Discharged
6	41/M	Endotracheal aspirate	Bronchopneumonia with respiratory failure	None	Tigecycline	Discharged
7	57/F	Endotracheal aspirate	Bronchopneumonia	Type II DM	Tigecycline	Discharged
8	31/M	Sputum	Aspirational pneumonia	None	Tigecycline	Discharged
9	62/F	Endotracheal aspirate	Type II Respiratory failure	HTN	Tigecycline	Discharged
10	9 days/M	Blood	Respiratory failure secondary to hyaline membrane disease	None	Tigecycline	Discharged
11	74/M	Sputum	Pneumonia	HTN Type II DM	Tigecycline	Discharged
12	70/F	Pus	Necrotizing fasciitis	HTN Type II DM	Tigecycline	Discharged
13	59/F	Catheter tip swab	Carcinoma pancreas; Severe Acute Respiratory Distress Syndrome with Type I respiratory failure	HTN	Tigecycline	Died
14	49/M	Endotracheal aspirate	Ventilator-associated Pneumonia (VAP), Acute Respiratory Distress Syndrome (known case of traumatic burst fracture, corpectomy)	Type II DM	Tigecycline	Died
15	71/M	Pus	Sepsis, Left Pleural effusion, Grade III bed sores	HTN Type II DM Iron Deficiency Anemia	Tigecycline	Discharged
16	85/M	Sputum	Bronchopneumonia	HTN Type II DM	Tigecycline	Discharged
17	62/F	Blood	Ischemic heart disease, Aspiration Pneumonia with septic shock	HTN Newly diagnosed DM Type II	Tigecycline	Died

Overall, tigecycline demonstrated favorable clinical outcomes in the majority of cases, particularly in respiratory and soft tissue infections, although mortality remained notable in critically ill patients with multiple comorbidities.

A total of 17 non-duplicate colistin-resistant *K. pneumoniae* isolates were included in this study, and antimicrobial susceptibility and synergy testing were performed using the disk elution method in CA-MHB. Results were interpreted according to CLSI M100 (35th edition, 2025) guidelines.

**Figure 2:**
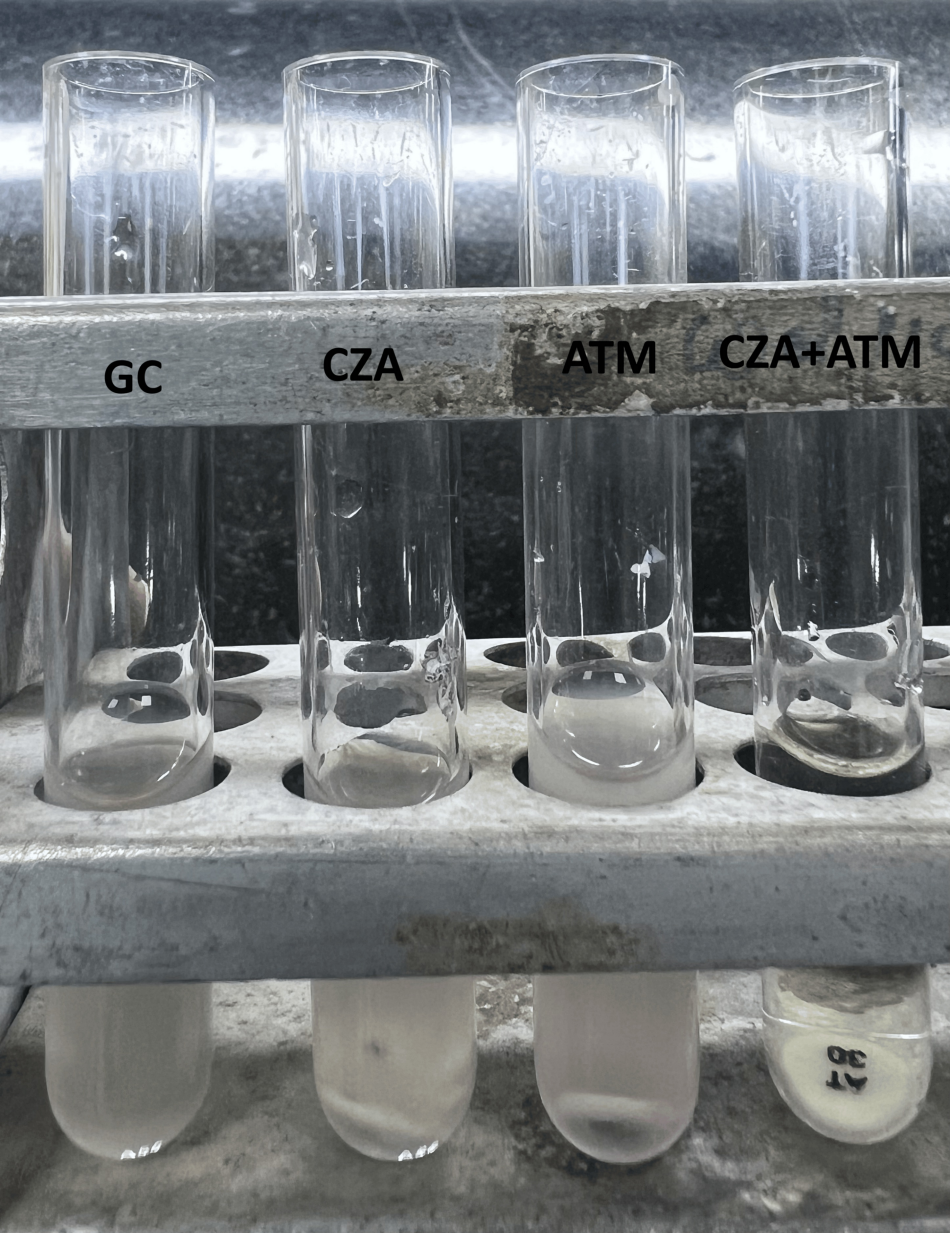
In vitro synergy testing of CZA and ATM against colistin-resistant CRKP CRKP: carbapenem-resistant *Klebsiella pneumoniae; *CZA: ceftazidime-avibactam; ATM: aztreonam; GC: growth control

Growth of the bacteria was assessed based on turbidity, where visible turbidity indicated bacterial growth (resistance) and the absence of turbidity indicated inhibition (susceptibility). Synergistic activity was defined as the absence of growth in the tube containing both antimicrobial disks (CZA and ATM) (Figure 2).

Susceptibility patterns

Out of the 17 isolates tested, 12 isolates (70.6%) demonstrated susceptibility only to CZA and ATM combination, indicating synergistic activity. Three isolates (17.6%) were susceptible to CZA alone, while two isolates (11.8%) were susceptible to ATM alone (Table 2). The majority of isolates exhibited a pattern consistent with synergistic susceptibility to the combination therapy, whereas a smaller proportion showed susceptibility to individual agents.

**Table 2:** Distribution of susceptibility patterns CZA: ceftazidime-avibactam; ATM: aztreonam

Susceptibility Pattern	Number (n = 17)	Percentage
Synergistic (CZA+ATM)	12	70.60%
CZA alone susceptible	3	17.60%
ATM alone susceptible	2	11.80%

## Discussion

The increasing prevalence of combined carbapenem and colistin resistance in *K. pneumoniae* is indicative of an evolving extensively drug-resistant and potentially pan-drug-resistant phenotype that poses a very serious therapeutic challenge in clinical practice. In the present study, 17 colistin-resistant CRKP isolates were evaluated, of which a substantial proportion (70.6%) demonstrated susceptibility to the combination of CZA and ATM. A smaller subset of isolates remained susceptible to CZA (17.6%) or ATM alone (11.8%). This distribution provides important insight into the underlying resistance mechanisms and their clinical implications.

The predominance of synergistic activity observed in this study strongly suggests that most isolates harbor multiple coexisting β-lactamase-mediated resistance mechanisms. In particular, the coexistence of MBLs, such as NDM, along with serine β-lactamases, including ESBLs and AmpC enzymes, is likely responsible for the observed resistance patterns. In scenarios like this, ATM alone is rendered ineffective due to degradation by ESBL and AmpC enzymes, while CZA alone is insufficient because avibactam lacks activity against MBLs. The restoration of antimicrobial activity with the combination of these drugs reflects the complementary mechanism of action of these agents, wherein avibactam protects ATM from enzymatic hydrolysis, thereby enabling it to act against MBL-producing organisms [10].

The findings of the present study are consistent with emerging regional and global data. Bakthavatchalam et al. (2022) reported that the combination of CZA and ATM restored susceptibility in a majority of CRKP isolates in South India, particularly those coharboring blaNDM and ESBL genes [11]. Similarly, Falcone et al. (2024) demonstrated high rates of microbiological clearance in bloodstream infections caused by MBL-producing organisms treated with this combination [12]. Furthermore, large-scale surveillance data from Sader et al. (2023) confirmed enhanced in vitro activity of the CZA-ATM combination against MBL-producing Enterobacterales across diverse geographic regions [13]. The concordance of the present findings with these studies reinforces the relevance of this combination therapy, particularly in MBL-endemic settings in India.

A smaller proportion of isolates (17.6%) in this study were susceptible to CZA alone, suggesting the absence of MBL production in these strains. In such cases, resistance is likely mediated by serine carbapenemases such as KPC and OXA-48, both of which are effectively inhibited by avibactam. This observation aligns with global epidemiological trends, where regions with a higher prevalence of KPC-producing organisms report greater efficacy of CZA monotherapy. Gou et al. (2023) reported favorable clinical outcomes with CZA in non-MBL-producing isolates, supporting its role as a targeted therapeutic option in such contexts [14]. However, in regions like South Asia, where MBL production predominates, reliance on CZA monotherapy remains limited.

Only a small fraction of isolates (11.8%) demonstrated susceptibility to ATM alone, representing a relatively uncommon resistance phenotype. This finding suggests that these isolates may produce MBL enzymes in the absence of significant co-expression of ESBL or AmpC β-lactamases, thereby allowing ATM to retain its activity. However, such isolates are infrequently encountered, as resistance determinants are often co-located on mobile genetic elements such as plasmids. Studies from India have similarly reported low rates of ATM susceptibility among CRKP isolates. Shanker et al. (2019) highlighted the rarity of isolated MBL production without accompanying β-lactamases, while Mishra et al. (2025) also observed limited effectiveness of ATM monotherapy in clinical isolates [15-16]. These findings underscore the limited reliability of ATM as a standalone agent and further highlight the need for combination therapy.

From a clinical point of view, the high rate of synergy observed in this study supports the potential role of CZA plus ATM as a carbapenem- and colistin-sparing therapeutic strategy. Given the well-recognized nephrotoxicity associated with polymyxins and the increasing incidence of resistance among these agents, alternative regimens with improved safety and efficacy profiles are urgently required. Early identification of synergistic susceptibility through laboratory testing may facilitate the timely initiation of appropriate antibiotic therapy and improve clinical outcomes in patients with severe infections caused by extensively drug-resistant CRKP [17].

The present case series suggests that in patients whose isolates are susceptible to the CZA and ATM combination, this regimen would likely offer greater clinical benefit than tigecycline. CZA + ATM provides a more targeted and mechanistically robust approach against carbapenemase-producing Enterobacterales, particularly those harboring MBLs along with co-produced serine β-lactamases. This combination is more effective in severe infections such as bloodstream infection, ventilator-associated pneumonia, sepsis, and deep-seated hospital-acquired infections, where a more reliable bactericidal regimen is preferred for prompt microbiological clearance and clinical response. In contrast, tigecycline is often considered a salvage option and may be limited by concerns regarding low serum concentrations, variable activity in critically ill patients, and less dependable efficacy in invasive infections. Therefore, demonstration of susceptibility to CZA + ATM supports a shift from empiric salvage therapy to a more effective, mechanism-based treatment strategy, with the potential to improve outcomes and strengthen antimicrobial stewardship.

From an antimicrobial stewardship standpoint, these findings highlight the importance of microbiologically guided therapy. As such, empirical use of novel antimicrobial combinations without confirmatory testing may lead to the emergence of resistance. Incorporation of simple and feasible methods such as disk elution for synergy testing, as utilized in our study, can provide valuable guidance for clinicians, particularly in resource-limited settings where advanced molecular diagnostic methods may not be readily available.

Despite its clinical relevance, the present study has certain limitations. The relatively small sample size may limit the generalizability of the findings. In addition, molecular characterization of resistance genes was not performed, which further restricts the precise correlation between phenotypic patterns and the underlying genotypes.

Subsequent investigations ought to emphasize the amalgamation of phenotypic and molecular methodologies to enhance the characterization of resistance mechanisms. To confirm the therapeutic effectiveness of this combination, larger multicenter studies that include clinical outcome data are necessary. Pharmacokinetic and pharmacodynamic research may also help optimize dosing regimens for CZA and ATM, making them more useful in clinical practice.

## Conclusions

The present study demonstrates that a considerable proportion of colistin-resistant *K. pneumoniae* isolates exhibited restored susceptibility when exposed to the combination of CZA and ATM, with synergistic activity observed in 70.6% of cases. This finding highlights the ability of this combination to effectively overcome complex resistance mechanisms, particularly in settings where MBL-producing strains are prevalent.

The observed synergy underscores the therapeutic potential of this regimen as a carbapenem- and colistin-sparing option in the treatment of infections caused by extensively drug-resistant CRKP. Given the limited efficacy and toxicity concerns associated with conventional last-line agents, this combination provides a rational and potentially safer alternative, especially in cases where standard therapies fail. Although the results are based on in vitro observations, they offer important preliminary evidence supporting the use of CZA with ATM in MBL-producing isolates that are resistant to both carbapenems and colistin.
